# The association of vitamin D deficiency, age and depression in US adults: a cross-sectional analysis

**DOI:** 10.1186/s12888-023-04685-0

**Published:** 2023-07-24

**Authors:** Hongfei Mo, Jipeng Zhang, Chiwei Huo, Mengying Zhang, Jiang Xiao, Junge Peng, Guirong Wang, Changhong Wang, Yan Li

**Affiliations:** 1 Synergetic Innovation Center of Kinesis and Health, School of Physical Education (Main Campus), Zhengzhou University, Zhengzhou, Henan P.R. China; 2 Zhengzhou University, Zhengzhou, Henan P.R. China; 3 Departments of Surgery and Microbiology & Immunology, SNUY Upstate Medical University, Syracuse, NY USA; 4 The Second Affiliated Hospital of Xinxiang Medical University, Xinxiang, Henan P.R. China

**Keywords:** Age, Depression, Vitamin D Deficiency, NHANES

## Abstract

**Background:**

Depression is an important public health burden, its risk of occurrence is associated with vitamin D deficiency and may also increase with age, while serum vitamin D levels are closely related to age.

**Objective:**

The purpose of this study was to evaluate whether vitamin D and age are associated with depression after adjustment for each other.

**Materials and methods:**

We extracted data from NHANES 2013–2018, including demographic characteristics, depression level, vitamin D level, physical activity, and body measures. A total of 15,156 adults aged 20 years or older (mean age 49.81 ± 17.67 years, 7301 males and 7855 females) were included. Depression was screened by PHQ-9. Vitamin D deficiency was defined by a serum vitamin D level < 30nmol/L. We performed binary logistic regression models to analyze the association between vitamin D, age and depression, respectively.

**Results:**

Vitamin D levels were negatively associated with depression (*P* < 0.001). Vitamin D had a significant effect on depression (OR = 0.776, 95%CI: 0.682–0.884, *P* < 0.001), the effect remained significant after adjusted for confounding variables (OR = 0.761, 95%CI: 0.663–0.874, *P* < 0.001). Age was positively associated with depression (*P* < 0.001) and had a significant effect on depression (OR = 1.079, 98%CI: 1.032–1.128, *P* = 0.001), the effect remained significant after adjusted for confounding variables (OR = 1.092, 95%CI: 1.040–1.146, *P* < 0.001). Age and vitamin D levels were positively correlated (*P* < 0.001), and older age had a significant effect on vitamin D level (OR = 1.526, 95%CI: 1.416–1.645, *P* < 0.001), the effect remained significant after adjusted for confounding variables (OR = 1.371, 95%CI: 1.263–1.487, *P* < 0.001). In addition, the prevalence of depression was higher in females (2312/7855, 29.43%) than in males (1571/7301, 21.52%), and the difference was statistically significant (*P* < 0.001).

**Conclusions:**

Vitamin D deficiency and older age are both associated with higher risk of depression, while older age is a protective factor for vitamin D deficiency.

## Background

Depression is one of the most common mental disorders worldwide, which has become a major public health concern [1], and one of the leading causes of the global burden of disease [2]. Depression is a widespread chronic mental illness that affects thoughts, mood and physical health, and it is characterized by low mood, lack of energy, sadness, insomnia and low self-esteem [3]. Epidemiological investigations showed that over 350 million people had suffered from depression in 2010 [4], with an incident rate of up to 25% in females and 12% in males [4, 5]. Hasin predicted that the 12-month and lifetime prevalence of depression was 10.4% and 20.6% in Americans [6]. However, the situation continues to deteriorate, and recent surveys showed that the occurrence of depression in U.S. adults had increased [7, 8]. It is urgent to discover the risk factors of depression and formulate intervention measures.

Recent evidences suggested that poor vitamin D status were often observed in patients with depression [9]. Vitamin D deficiency has a high global incidence and is considered to be associated with an increased risk of major depression and anxiety [10]. Major clinical studies have shown that vitamin D supplementation can help alleviate symptoms of depression [10]. Depression affects 3% of the elderly population and 10–20% of the elderly patients with chronic conditions, which is an important later-life health concern [11]. If not treated timely, depression in the elderly may lead to serious limb disabilities, suicides and poor outcome of diseases, leading to death [11]. Compared with the younger populations, the older populations may be more likely to suffer from depression, and their situations could be sever.

However, the existing studies mainly focused on the relationship between vitamin D and depression, or the relationship between age and depression, lacking analysis and comparison of the relationship between these three predictors in the same sample. In addition, there is also a lack of large sample epidemiological surveys in these three predictors. Therefore, it is particularly necessary to conduct reliable cross-sectional analysis of large samples in a representative population. The purpose of this study was to evaluate whether vitamin D and age are associated with depression after adjustment for each other through rigorous analysis and comparison of samples from a large eligible population.

## Materials and methods

### Study Population

**Fig. 1:**
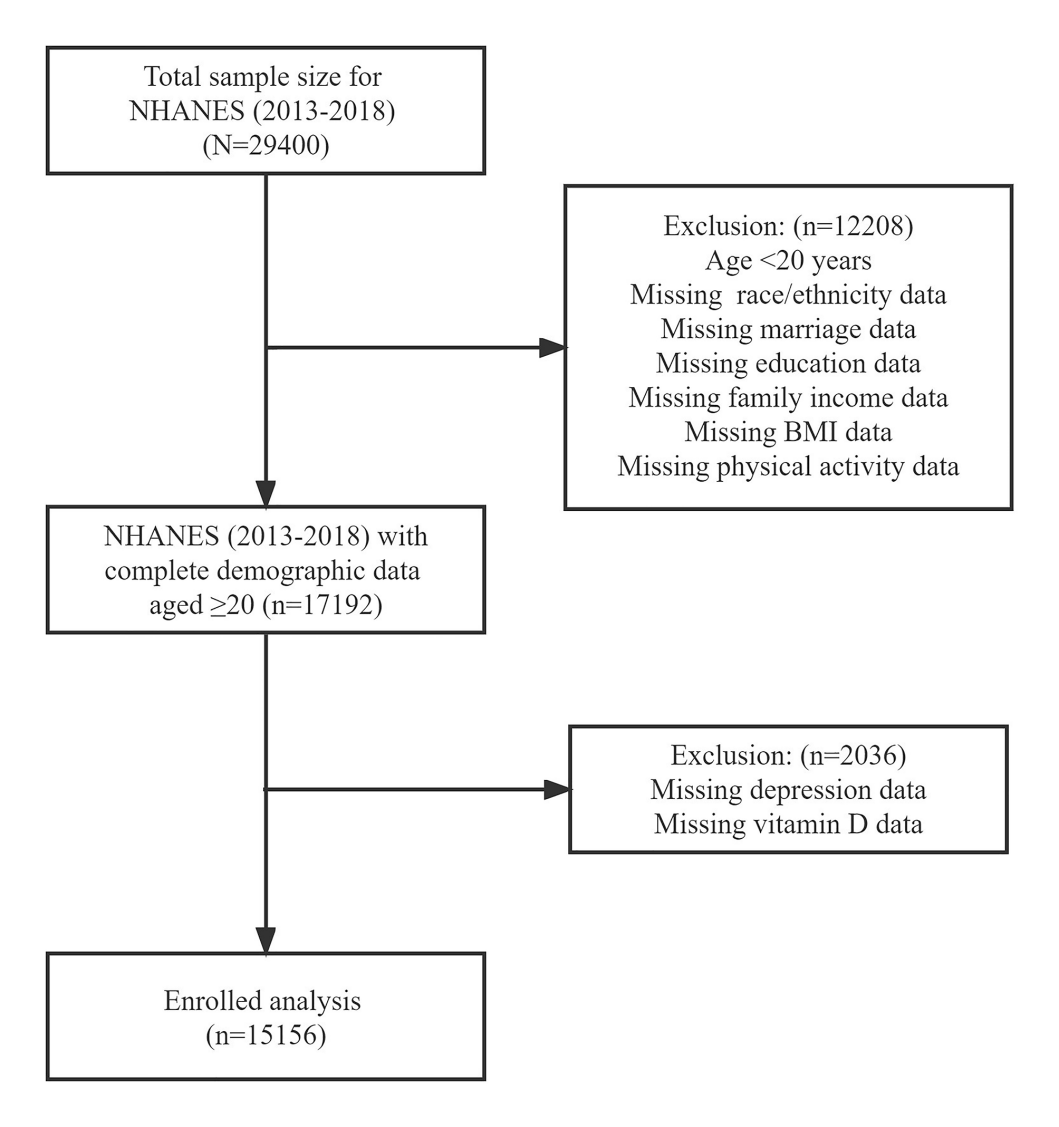
Flow chart of subject selection

The National Health and Nutrition Examination Survey (NHANES) is a population-based cross-sectional survey designed to collect information about the health and nutrition situation of the U.S. household population [12]. A stratified multistage sampling design was used to obtain a representative sample of U.S. residents of two months or older; the two-year survey cycle covers about 15,000 families. The NHANES protocol was approved by the National Center for Health Statistic’s research ethics review board; all adult participants provided written notice of consent [13]. The present study extracted and aggregated data on depression, vitamin D, body measures, other questionnaire data and demographic characteristics from the NHANES 2013–2018, and the current sample is restricted to adults aged 20 and older. The total sample size assessed for the study was 15,156, and additional details about the study design, sampling and exclusion criteria are presented in the figure below. (See Fig. 1)

### Depression Assessment

The Patient Health Questionnaire (PHQ-9), a nine-item depression screening instrument, was used to assess the frequency of depression symptoms in the sample over the past 2 weeks. The questions were asked at the Mobile Examination Center (MEC) by trained interviewers using the Computer-Assisted Personal Interview (CAPI) system, which is programmed with built-in consistency checks to reduce data entry errors as part of the MEC interview. For each item, points ranging from 0 to 3, are associated with the response categories “not at all”, “several days”, “more than half the days”, and “nearly every day”. [14, 15]. A total score, ranging from 0 to 27, can be calculated for persons with complete responses to the symptom questions. Those who scored less than 5 were considered to have no depression; those who scored 5–9 were classed as mild depression; those who scored 9–19 were classed as moderate depression; and those who scored more than 19 were classed as severe depression. In this study, PHQ-9 scores of 0–4 were classed as the “Non-depressed group’ and scores of 5–27 were classed as the “Depressed group”[15]. Moreover, the last question: “Over the last 2 weeks, how often have you been bothered by the following problem: Thoughts that you would be better off dead or of hurting yourself in some way?” was used to assess participants’ suicidal attempt [12].

### Vitamin D measurement

Vitamin D level was measured through methods from CDC: the test principle used high-performance liquid chromatography tandem mass spectrometry (HPLC-MS/MS) to quantitatively detect 25-hydroxyvitamin D3 (25OHD3), 3-epi-25-hydroxyvitamin D3 (epi-25OHD3) and 25-hydroxyvitamin D2 (25OHD2) in human serum. In this study, the level of 25-hydroxyvitamin D2 plus 25-hydroxyvitamin D3 was used to express the vitamin D content. Measured in nmol/L, 30 nmol/L was used as the classification standard of vitamin D content: level < 30 nmol/L was classed as vitamin D deficiency, level ≥ 30 nmol/L was classed as moderate to excessive level (no deficiency)[16]. Code “0” and “1” were ranked to represent vitamin D deficiency and no deficiency in the final database, respectively.

### Covariates

Covariates of this study included gender, age, race/ethnicity, education, marital status, income to poverty, body mass index (BMI) and physical activity. A total of 15,156 participants were categorized into three age groups: 20–39, 40–59, and ≥ 60. Race/ethnicity was categorized as Hispanic, non-Hispanic White, non-Hispanic Black, non-Hispanic Asian and Other. Educational was categorized as Below high school, High school, and Post high school. Marital status was categorized as Cohabiting, Married living alone (widowed, divorced, separated,) and Unmarried. income to poverty was calculated by dividing family income by the poverty guidelines for the survey year. Poverty guidelines vary by family size and geographic location [17]. For this study, PIR was used to create two categories of income status, impoverished (< 1.3) and moderate income (≥ 1.3)[18]. During the MEC physical examination, weight and height were measured by trained technicians using standardized equipment, BMI was categorized into four standard categories in the raw data: Underweight (≤ 18.9 kg/m^2^), Normal weight (19.0-24.9 kg/m^2^), Overweight (25.0-29.9 kg/m^2^), and Obese (≥ 30.0 kg/m^2^) [19]. Physical activity was assessed through a Global Physical Activity Questionnaire (GPAQ), which classifies physical activity into four components: work, traffic, recreational physical activity, and sedentary behavior. In addition to sedentary behavior, those who participated in only one moderate intensity physical activity were classed as “Insufficiently active”, those who participated in more than one moderate or higher intensity physical activities were classed as “Sufficiently active”, and those who did not participate were classed as “Inactive”.

### Statistical analyses

We first collected the extracted raw data using Microsoft Excel 2010 and excluded missing and useless (refused, not known) items. Adults aged 20 years and older with complete information of depression, vitamin D, age, BMI and other demographic characteristics were included in the database. In accordance with the purpose of this study, we analyzed descriptive characteristics separately for the depression and non-depression groups. Chi-square tests or Kruskal-Wallis tests were performed for mono-factor analysis of count variables, while t-tests were performed for significance test of categorical variables. Binary logistic regression was performed separately to analyze the association between vitamin D and depression, age and depression, vitamin D and age. Variables that were statistically significant in the mono-factor analysis were included in the logistic stepwise regression analysis. A-entry = 0.05 and a-exit = 0.10 were used to select and exclude independent variables.

In analysis for the association of vitamin D and depression, we confirmed vitamin D as the independent variable (0 = deficiency, 1 = no deficiency) and depression (0 = no depression, 1 = depression) as the dependent variable. In order to exclude the influences of confounding variables, the following models were developed: Model I: Original model, no adjustment of any variables. Model II: Adjusted for the independent variables in Model I plus demographic characteristics (gender, race, education, and marital status). Model III: adjusted for variables in Model II plus suicidal attempt. Model IV: adjusted for variables in Model III plus age.

In analysis for the association age and vitamin D, we confirmed age as the independent variable (“1”=20–39 years, “2”=40–59 years, “3”=60 years and older) and vitamin D as the dependent variable (0 = deficient, 1 = not deficient).To exclude the influence of confounding variables, the following models were developed: Model V: Original model, no adjustment of any variables. Model VI: Adjusted for the independent variables in Model V plus demographic characteristics (gender, race, education, and marital status). Model VII: adjusted for the independent variables in Model VI plus suicidal attempt.

In analysis for the association of age and depression, we also confirmed age as the independent variable (“1”=20–39 years, “2”=40–59 years, “3”=60 years and older) and depression as the dependent variable (0 = non-depression, 1 = depression).To exclude the influence of confounding variables, the following models were developed: Model VIII: Original model, no adjustment of any variables. Model IX: adjusted for the independent variables in Model VIII plus demographic characteristics (gender, race, education, and marital status). Model X: adjusted for the independent variable in Model IX plus vitamin D. Model XI: adjusted for the independent variable in Model IX plus suicidal attempt. All data were analyzed using the Statistical Package for Social Sciences (SPSS) version 28.0, p-values less than 0.05 were considered statistically significant (two-sided test).

## Results

### Demographic characteristics

This study included a total of 15,156 adults aged 20 years or older in the NHANES cycle 2013–2014, 2015–2016 and 2017–2018 who have completed data on vitamin D level, degree of depression, and other demographic information. Mean age of participants was 49.81 ± 17.67 years at the time of examination, including 7301 males and 7855 females, with a mean vitamin D level 66.52 ± 29.50 nmol/L. Significant statistical differences were observed (*P* < 0.001) in different gender, age, race, education, marital status, income to poverty, BMI, suicidal attempt and vitamin D between the depression group and the non-depression group. Differences were not observed in physical activity (*P* = 0.221). (See Table 1)

**Table 1:** Demographic characteristics of NHANES 2013-18 adults aged ≥ 20 by depression

Characteristics, n%	Sample Capacity		Depression		Non-depression		Test statistics	P
	N = 15,156		n = 3883		n = 11,273			
**Gender**							124.424^a^	< 0.001^***^
Male	7301	(48.17)	1571	(40.46)	5730	(50.83)		
Female	7855	(51.83)	2312	(59.54)	5543	(49.17)		
**Age Group**							-9.827^b^	< 0.001^***^
20–39	5031	(33.19)	1189	(30.62)	3842	(34.08)		
40–59	4979	(32.85)	1328	(34.20)	3651	(32.39)		
≥ 60	5146	(33.95)	1366	(35.18)	3780	(33.53)		
**Race**							-5.201^b^	< 0.001^***^
Hispanic	3642	(24.03)	973	(25.06)	2669	(23.68)		
Non-Hispanic White	5814	(38.36)	1588	(40.90)	4226	(37.49)		
Non-Hispanic Black	3215	(21.21)	832	(21.43)	2383	(21.14)		
Non-Hispanic Asian	1894	(12.50)	311	(8.01)	1583	(14.04)		
Other	591	(3.90)	179	(4.61)	412	(3.65)		
**Education**							-11.479^b^	< 0.001^***^
Below high school	3154	(20.81)	1021	(26.29)	2133	(18.92)		
High school	3458	(22.82)	958	(24.67)	2500	(22.18)		
Post high school	8544	(56.37)	1904	(49.03)	6640	(58.90)		
**Marital Statues**							184.221^a^	< 0.001^***^
Cohabitation	9010	(59.45)	1973	(50.81)	7037	(62.42)		
Married living alone	3381	(22.31)	1125	(28.97)	2256	(20.01)		
Not married	2765	(18.14)	785	(20.22)	1980	(17.56)		
**Income to Poverty**							238.087^a^	< 0.001^***^
Impoverished	4825	(31.84)	1623	(41.80)	3202	(28.40)		
Moderate income	10,331	(68.16)	2260	(58.20)	8071	(71.60)		
**BMI**							-9.103^b^	< 0.001^***^
Underweight	479	(3.16)	132	(3.40)	347	(3.08)		
Normal weight	3932	(25.94)	863	(22.23)	3069	(27.22)		
Overweight	4744	(31.30)	1080	(27.81)	3664	(32.50)		
Obese	6001	(39.59)	1808	(46.56)	4193	(37.20)		
**Physical Activity**							-1.224^b^	0.221
Inactive	8794	(58.02)	2217	(57.10)	6577	(58.34)		
Insufficiently active	2962	(19.54)	784	(20.19)	2178	(19.32)		
Sufficiently active	3400	(22.43)	882	(22.71)	2518	(22.34)		
**Suicidal Attempt**							1354.514^a^	< 0.001^***^
Yes	559	(3.69)	516	(13.29)	43	(0.38)		
No	14,597	(96.31)	3367	(86.71)	11,230	(99.62)		
**Serum Vitamin D**							14.744^a^	< 0.001^***^
< 30nmol/L	1185	(7.82)	359	(9.25)	826	(7.33)		
≥ 30nmol/L	13,971	(92.18)	3524	(90.75)	10,447	(92.67)		

^*a*^ *chi-square test*, ^*b*^ *Kruskal-Wallis-test*, ^*^P < 0.05, ^**^P < 0.01, ^***^P < 0.001, same as below; Depression: PHQ-9 score ≥ 5, non-Depression: PHQ-9 score < *5; BMI: Body Mass Index; Vitamin D Deficiency: Serum Vitamin D < 30nmol/L*

### Depression percentage in this study

More than a quarter of the samples were detected as depression individuals in this study. In a total of 15,156 samples, 11,273 (74.38%) participants with a PHQ-9 score < 5 were classed as no depression individuals; 3883 (25.62%) participants with a PHQ-9 score ≥ 5 were classed as depression individuals, among which 2521 (16.63%) had mild depression, and 1362 (8.99%) had moderate to severe depression [1211 (7.99%) for moderate, and 151 (1.00%) for severe, respectively] which were generally believed to require professional psychiatric treatments. (See Table 2)

**Table 2:** Depression status in NHANES 2013-18 adults aged ≥ 20

PHQ-9 Score	Depression Status	n	Percentage (%)
0–4	no depression	11,273	74.38
5–9	mild depression	**2521**	**16.63**
10–19	moderate depression	**1211**	**7.99**
20–27	severe depression	**151**	**1.00**

### Logistic regression analysis

#### Association of vitamin D and depression

In this analysis, gender, race, education, marital status, age, vitamin D and suicidal attempt were included in the regression models. Model I (without excluding any confounders) showed an odds ratio (OR) of 0.776 (95% CI: 0.682–0.884) for the association of vitamin D with depression; Model II (adjusted for variables of gender, race, education, and marital status) showed an OR = 0.804 (95% CI: 0.704–0.918), Model III (adjusted for suicidal attempt) showed OR = 0.777 (95% CI: 0.678–0.891); Model IV (adjusted for age) showed OR = 0.761 (95% CI: 0.663–0.874). The results suggest that vitamin D deficiency remains a risk factor for depression after adjusted for confounders. Those with vitamin D deficiency are more likely to develop depression. (all *P* < 0.01)(See Table 3).

**Table 3:** Weighted association of vitamin D and depression in NHANES 2013-18 adults aged ≥ 20

Mode	*b*	SE	*Wald*	*P*	OR(95%CI)
I^c^	-0.253	0.066	14.680	< 0.001^***^	0.776(0.682–0.884)
II^d^	-0.218	0.067	10.450	0.001^**^	0.804(0.704–0.918)
III^e^	-0.252	0.070	12.988	< 0.001^***^	**0.777(0.678–0.891)**
IV^f^	-0.273	0.070	15.068	< 0.001^***^	**0.761(0.663–0.874)**

^*c*^ *Original model, no adjustment of any variables.* ^*d*^ *Adjusted for the independent variable in Model* I *plus gender, race, education and marital status.* ^*e*^ *Adjusted for the independent variable in Model* II *plus suicide attempt.* ^*f*^ *Adjusted for the independent variable in Model* III *plus age.*

#### Association of age and vitamin D

In this analysis, gender, race, education, marital status, age and suicidal attempt were included in the regression models. Model V (without excluding any confounders) showed an OR = 1.526 (95% CI: 1.416–1.645) for the association of age and vitamin D non-deficiency; Model VI (adjusted for gender, race, education, and marital status) showed an OR = 1.371 (95% CI: 1.263–1.488), and Model VII (adjusted for suicidal attempt) showed OR = 1.371 (95% CI: 1.263–1.487). The results showed that older age remained a protective factor for vitamin D deficiency after adjusted for confounders. For every 20-year decrease in age, the prevalence of vitamin D deficiency increased 1.371-fold. (all *P* < 0.01)(See Table 4).

**Table 4:** Weighted association of age and vitamin D in NHANES 2013-18 adults aged ≥ 20

Mode	*b*	SE	*Wald*	*P*	OR(95%CI)
V^g^	0.423	0.038	122.014	< 0.001^***^	1.526(1.416–1.645)
VI^h^	0.316	0.042	57.247	< 0.001^***^	1.371(1.263–1.488)
VII^i^	0.315	0.042	57.102	< 0.001^***^	**1.371(1.263–1.487)**

^*g*^ *Original model, no adjustment of any variables.* ^*h*^ *Adjusted for the independent variable in Model* V *plus gender, race, education and marital status.* ^*i*^ *Adjusted for the independent variable in Model* VI *plus suicide attempt.*

#### Association of Age and Depression

In this analysis, gender, race, education, marital status, age, vitamin D and suicidal attempt were included in the regression models. Model VIII (without excluding any confounders) showed an OR = 1.079 (95% CI: 1.032–1.128) for the association of age and depression; model IX (adjusted for gender, race, education, and marital status) showed an OR = 1.095 (95% CI: 1.045–1.146), model X (adjusted for vitamin D) showed an OR = 1.102 (95% CI: 1.052–1.154), and model XI (adjusted for suicidal attempt) showed OR = 1.092 (95% CI: 1.040–1.146). The results showed that older age remained a risk factor for developing depression after adjusted for confounders. For each 20-year increase in age, the prevalence of developing depression increased 1.092-fold. (all *P* < 0.01). (See Table 5)

**Table 5:** Weighted association of age and depression in NHANES 2013-18 adults aged ≥ 20

Mode	*b*	SE	*Wald*	*P*	OR(95%CI)
VIII^j^	0.076	0.023	11.218	0.001^**^	1.079(1.032–1.128)
IX^k^	0.090	0.024	14.748	< 0.001^***^	1.095(1.045–1.146)
X^l^	0.097	0.024	16.874	< 0.001^***^	**1.102(1.052–1.154)**
XI^m^	0.088	0.025	12.692	< 0.001^***^	**1.092(1.040–1.146)**

^*j*^ *Original model, no adjustment of any variables.* ^*k*^ *Adjusted for the independent variable in Model* VIII *plus gender, race, education and marital status.* ^*l*^ *Adjusted for the independent variable in Model* IX *plus vitamin D.* ^*m*^ *Adjusted for the independent variable in Model* X *plus suicidal attempt.*

## Discussion

Through Logistic Regression analysis of data from the NHANES 2013–2018, we found that vitamin D deficiency was associated with an increased risk of depression; older age was associated with an increased risk of depression; and older age was a protective factor for maintaining normal vitamin D level. These findings were derived from independent analyses of these three variables. Therefore, we will discuss “vitamin D deficiency and depression”, “age and vitamin D”, and “age and depression”, respectively, in the following text.

### Vitamin D Deficiency and Depression

The results of this study support previous related researches. Lower vitamin D levels were associated with increased symptoms of depression and anxiety [20]. Regression results from the present study suggest that those who obtain moderate vitamin D levels are 76.1% as likely to develop depression than those who are deficient, and that maintaining above-normal levels of serum vitamin D levels is a protective factor against depression which may be an effective means of preventing depressive disorders.

Vitamin D may mediate effects on depression via calcium ions (Ca^2+^) and 5-Hydroxytryptamine (5-HT). Vitamin D acts on human cells and maintains their internal Ca^2+^ concentration at a low level, so when vitamin D levels fall, Ca^2+^ levels in brain cells rise, leading to an increased risk of depression. Vitamin D also plays a role in maintaining normal 5-HT synthesis and induces the demethylases expression of genetic materials. Normally, a normal vitamin D level promotes normal DNA transcription, maintains normal neural activity, and thus prevents depression [21].

In addition, solar exposure and low calcium intake are likely to be indirect factors affecting depression. Vitamin D deficiency has been shown to be significantly associated with exposure to sunlight. Major global vitamin D deficiency may be strongly related to public underestimation of the role of sunlight in providing vitamin D requirements for human body; vitamin D is mainly produced through biosynthesis, and few foods naturally contain, while sunlight exposure is practically essential to initiate the synthetic reaction [22]. One of the major functions of vitamin D is to promote calcium absorption, however, low calcium intake can disrupt this regulatory mechanism, before exacerbating the consequences of vitamin D deficiency [23].

### Age and vitamin D

Younger populations may be more susceptible to vitamin D deficiency due to metabolic factors and physical activity. This study showed a significant association between age and vitamin D. Regression models demonstrated that for every 20-year decrease in age, the risk of vitamin D deficiency increased by 37.1%.

Previous studies [24–26] have mostly suggested that older people are more susceptible to vitamin D deficiency, and our findings do not support this view. The reason for this may be that the findings of previous studies are mostly secondary endpoints in studies of vitamin D deficiency in the elderly, women or adolescents, where there has been specific national coverage of epidemiological surveys, or there where confounders not been excluded as in the present study.

However, there is also view contrary to previous studies which support our finding. Evidences indicated that depression in young people had increased in the UK and may be related to the age-specifics characteristics of vitamin D [21]. There were studies suggested that younger individuals may be more susceptible to vitamin D deficiency, which may be due to better metabolic capacity than in older individuals [27]. Normal levels of vitamin D maintain the processes of aging (such as autophagy, mitochondrial dysfunction, inflammation, oxidative stress, epigenetics and calcium alterations) at a normal low rate, which slows down the aging process. In this regard, younger people have a faster metabolism, while the aging process is carried out relatively at a lower rate. We believe that due to this mechanism, younger people may mobilize and utilize more vitamin D, which may result in a greater risk to vitamin D deficiency. One relevant study also indicated that vitamin D deficiency were frequently observed in professional athletes, with up to 90% presented deficient [28]. Relatively, younger people typically preformed greater physical activity, which may be one of the key reasons why younger people are more prone to vitamin D deficiency [29]. From this point of view, vitamin D deficiency are to be seriously concerned: younger individuals are more likely to be vitamin D deficient, and their physio-psychological need for vitamin D may also be higher.

### Age and Depression

Senior studies have shown that older people are more likely to suffer from depression [30–32], and the results of this study support previous studies. The prevalence of depression increases by 9.2% for every 20-year increase in age, which may be related to a variety of bio-physiological, psychological and sociological factors.

The physiological degeneration that occurs in older adults is associated with depression. It has been shown that decrements in neurological and psycho-physiological functions are closely related to aging [33]. Recent studies have shown that depression is associated with pain in older adults [34]. Vascular lesions in selected regions of the brain may contribute to a distinctive form of late-life depression, in addition to which endocrine changes have been associated [35]. Non-suppression of cortisol is associated with depression [36], which is associated with excessive secretion of corticotropin releasing factor (CRF): CRF are believed to mediate sleep and appetite disturbances, hypoesthesia and psychomotor changes [37], while responsiveness of adrenocorticotropic hormone, cortisol and dehydroepiandrosterone sulfate to CRF increased responsiveness to CRF are considered to be associated with aging [38].

Depression in older populations may be related to psychodynamics due to life experiences. Behavioral, psychodynamic, and cognitive aberrations are considered to be responsible for depression in older populations [38]. Depression is associated with emotional abuse and neglect during childhood, as well as behavioral problems and stress from primary social relationships in later adulthood [39]. In a meta-analysis, the number of total negative life events and daily hassles were associated with depression in an older population [40]. Negative achievement events were also associated with depression in those who place a high value on personal success and control [35]. Older individuals experienced more of these aspects, while younger individuals experienced fewer.

Increased social isolation may also be an influencing factor for depression in older populations. Associations between depression and impaired social support in order population has been established for years, impaired social support is associated with poor outcomes of depression in older males, but not in females [35]. Related studies have indicated that loneliness may be a key factor for depression [41]. After excluded confounding factors, older people who are less socially engaged were found to be more depressed (e.g. elderly people who stopped driving are at greater risk of worsening depressive symptoms) [42], whereas younger people always did better on these dimensions.

### Limitations of this study

This study has several limitations: (a) We have imperfections in excluding confounding factors: the etiology of depression is multi-factorial, including multiple genetic, bio-physiological, environmental and social factors, and this study failed to exclude all influencing factors. (b) This study extracted data from 2013 to 2018, but the emergence of the COVID-19 pandemic in 2020 raised many issues that directly or indirectly had a greater impact on the mental health of the general population, leading to changes in depression in the present post-epidemic era [19]. (c) Many related studies have been conducted prior to this study, challenging the innovative nature of the initial selection of this study. However, we have also made new discoveries. In our future design, we will consider avoiding these limitations and further explore the deeper mechanics of vitamin D and older age effecting depression based on the innovative points of this study, including not limited to biochemical and genetic studies.

## Conclusions

The results of this study suggested that vitamin D deficiency and older age are both associated with increased risk of depression, despite the fact that vitamin D deficiency was more prevalent in younger populations of our sample. These conclusions provide evidence for the view that “younger people are more likely to be vitamin D deficient” and contribute to scientific basis for early prevention of depression.

## Data Availability

The datasets generated and/or analysed during the current study are available in the [NHANES] repository, [NHANES Questionnaires, Datasets, and Related Documentation (cdc.gov)]. Raw data supporting the obtained results are available at the corresponding author.

## Abbreviations

NHANES: National Health and Nutritional Examination Survey
PHQ-9: The Patient Health Questionnaire
OR: Odds Ratio
CI: Confidence Interval
P: P-Value
U.S: The United States of America
MEC: Mobile Examination Center
CAPI: Computer-Assisted Personal Interview
CDC: Centers for Disease Control and Prevention
GPAQ: Global Physical Activity Questionnaire
HPLC-MS: High-Performance Liquid Chromatography Tandem Mass Spectrometry
25OHD3: 25-Hydroxyvitamin D3
25OHD2: 25-Hydroxyvitamin D2
BMI: Body Mass Index
SPSS: Statistical Package for Social Sciences
Ca^2+^: Calcium Ion
5-HT: 5-Hydroxytryptamine
DNA: Deoxyribo Nucleic Acid
CRF: Corticotropin Releasing Factor
COVID-19: Corona Virus Disease 2019
NCHS: National Center for Health Statistics.
